# Capacity and site readiness for hypertension control program implementation in Nigeria: A nationwide cross-sectional study

**DOI:** 10.1371/journal.pone.0344011

**Published:** 2026-03-13

**Authors:** Innocent Ijezie Chukwuonye, Ejiroghene Martha Umuerri, Abigail Baldridge, Yekeen Ayodele Ayoola, Mahmoud Umar Sani, Okechukwu Samuel Ogah, Olanike Allison Orimolade, Miracle Erinma Chukwuonye, Kalu Ulu Kalu, Caleb Ogechi Raphael, Nyemike Simeon Awunor, Obatavwe Ukoba, Felicity Chinyere Odoh, Henry Ifeanyichukwu Okolie, Mustapha Abdulsalam Danimoh, Apollos Daniel, Usman Muhammad Ibrahim, Sabiu Mohammed Hamza, Oluwatosin Zinnat Makinde, Adeyemi Sunday Adewole, Dike Bevis Ojji, Mark D. Huffman, on behalf of the Hypertension Treatment in Nigeria Program Investigators

**Affiliations:** 1 Department of Medicine, Faculty of Clinical Sciences, David Umahi Federal University of Health Sciences, Uburu, Nigeria; 2 Department of Internal Medicine, David Umahi Federal University Teaching Hospital, Uburu, Nigeria; 3 Department of Medicine, College of Health Sciences, Delta State University, Abraka, Nigeria; 4 Department of Medicine, Delta State University Teaching Hospital, Oghara, Nigeria; 5 Center for Dissemination and Implementation Science, Feinberg School of Medicine, Northwestern University, Chicago, United States of America; 6 Department of Internal Medicine, Federal Teaching Hospital, Gombe, Gombe, Nigeria; 7 Department of Medicine, Bayero University Kano, Nigeria; 8 Department of Medicine, Aminu Kano Teaching Hospital, Kano, Nigeria; 9 Department of Medicine, University of Ibadan, Ibadan, Oyo State, Nigeria; 10 Department of Medicine, University College Hospital, Ibadan, Oyo State, Nigeria; 11 Department of Family Medicine, Alex Ekwueme Federal University Teaching Hospital, Abakaliki, Ebonyi State, Nigeria; 12 Abia State Primary Health Care Department Agency, Umuahia, Abia State,; 13 Department of Public Health Nutrition, University of Port Harcourt, River State, Nigeria; 14 Department of Community Medicine, Delta State University Teaching Hospital, Oghara, Nigeria; 15 Department of Internal Medicine, Federal Teaching Hospital, Gombe State, Nigeria; 16 Department of Community Medicine, Gombe State University, Gombe, Nigeria; 17 Department of Community Medicine, Federal Teaching Hospital, Gombe State, Nigeria; 18 Department of Paediatrics, Federal Teaching Hospital, Gombe State, Nigeria; 19 Department of Community Medicine, Federal University Dutse, Jigawa State, Nigeria; 20 Department of Community Medicine, Rasheed Shekoni Federal Teaching Hospital, Dutse, Jigawa State, Nigeria; 21 Department of Internal Medicine, Federal University Dutse, Jigawa State, Nigeria; 22 Department of Internal Medicine, Rasheed Shekoni Federal Teaching Hospital, Dutse, Jigawa State, Nigeria; 23 Fountain Heart Clinic, Remilekun House, Ibadan, Oyo State, Nigeria; 24 Oyo State Primary HealthCare Development Agency, State Secretariat, Agodi Ibadan, Oyo State, Nigeria; 25 Department of Medicine, Faculty of Clinical Sciences, University of Abuja, Nigeria; 26 Department of Medicine, University of Abuja Teaching Hospital, Gwagwalada, Abuja, Nigeria; 27 Department of Medicine and Global Health Center, Washington University, St. Louis, Missouri, United States of America; 28 School of Public Health, Washington University in St Louis, Missouri, United States of America; 29 The George Institute for Global Health, University of New South Wales, Sydney, Barangaroo, Australia; Federal University Otuoke, NIGERIA

## Abstract

**Background:**

The aim of this study is to determine the capacity and readiness of Nigerian primary healthcare facilities to adopt a multi-level approach for the diagnosis, treatment, and control of hypertension.

**Methods:**

Using a multi-stage sampling technique, 5 states were selected for the nationwide study, and 10 Primary Healthcare Centres (PHCs) were selected from each of the participating states. The 50 PHCs were evaluated using the World Health Organization-modified Service Availability and Readiness Assessment, focusing on the diagnosis and treatment of hypertension in Nigeria. The indicator scores for general and cardiovascular service preparedness were computed using the proportion of PHCs with accessible facilities, tools, diagnostic guidelines, and prescription drugs.

**Results:**

A majority of PHCs (n = 43; 86%) reported having two or more full-time staff. The median number of full-time employees for the 50 PHCs was 6 (IQR = 2–9), and for the community health extension workers (CHEWs), the median was 2, the interquartile range (IQR) = 0–4. None of the PHCs had full-time physicians. Ninety-eight percent, 94%, and 84% of the 50 PHCs are able to provide screening services, diagnose, and confirm hypertension, respectively. In addition, 98% of the PHCs had functional blood pressure apparatus. However, only a minority of PHCs had the guidelines (24%), treatment algorithms (27%), and facilities. Most of the 50 PHCs (96%) use electronic patient records in their respective centres. Of the 50 PHCs studied, 66% had at least one 30-day antihypertensive treatment regimen in stock. The most commonly available drug classes were calcium channel blockers (72%), followed by diuretics (42%), central acting agents (38%), and angiotensin-converting enzyme inhibitors (36%). The median number of 30-day regimens in stock was 15 (IQR 0–132).

**Conclusion:**

This first large-scale systematic assessment of capacity and readiness for a system-level hypertension control program within five states of Nigeria demonstrated implementation feasibility based on the workforce, equipment, and health information systems, but there is a critical need for health-worker training and provision of protocols for hypertension treatment and control, as well as some need to strengthen the essential medicine supply chain.

## Introduction

Over 1.3 billion individuals are living with hypertension, and it remains the leading cause of death globally, accounting for about 10.4 million deaths per year. [1,2] Hypertension is a major independent risk factor for the development of renal failure, stroke, and coronary artery disease and is the leading cause of disability worldwide. [3,4] Approximately three-quarters of adults living with hypertension live in Africa and other developing countries with limited health resources and very low awareness of hypertension among the general population. [4,5] In 2019, over one billion people—representing 82% of the global hypertensive population—were living in low- and middle-income countries (LMICs). This marked a significant increase from 1990, largely driven by population growth, ageing, and a steady or rising prevalence of hypertension. Despite this growing burden, treatment and control rates in many LMICs remain alarmingly low. In some countries, fewer than 25% of women and less than 20% of men received treatment, while control rates for both sexes fell below 10%. [6] In addition, in 2019, the age-standardized prevalence of hypertension among adults aged 30–79 years in Nigeria was 36% (females: 39%, males: 33%), which is higher than the global average (33%). [7] Of the 19.1 million Nigerian individuals with hypertension, 47% have been diagnosed, 27% are receiving treatment, and merely 11% have achieved control of their hypertension. These rates are far below global averages (54% diagnosed, 42% treated, 21% controlled).

Primary healthcare is the most inclusive, equitable, cost-effective, and efficient approach to enhancing the physical and mental health of the general population. The majority of Nigeria’s primary healthcare centres (PHCs) are publicly owned, and the country currently has a network of slightly under 33,000 active PHCs, accounting for 85% of all medical facilities in the country, according to the Federal Ministry of Health, Health Facility Registry. [8] In order to incorporate hypertension service delivery into PHCs in the Federal Capital Territory, the Hypertension Treatment in Nigeria Program was established in 2019. The programme utilizes a type 2 hybrid, interrupted time series design across 60 PHCs, incorporating strategies such as patient registration, simplified treatment guidelines, and team-based care. [9] Baseline data revealed high treatment rates (89.2%) but low control rates (13.1%) among 4,927 participants.[10] By December 2023, treatment rates exceeded 90%, while control rates surpassed 50%. [11] These results offer important new information for better managing hypertension in Nigeria and other LMICs. It is crucial to remember that residents’ participation in PHC activities in local government areas is sine qua non for achieving the objectives of these PHCs. [12,13] PHCs provide the most viable route towards achieving health-related sustainable development goals (SDGs) and are crucial to the achievement of other SDGs [14].

However, one major concern that public PHCs in low- and middle-income countries (LMICs) frequently have is the lack of a system capacity for hypertension screening, diagnosis, registration of diagnosed patients, follow-up, provision of essential drugs, and treatment protocols for hypertension management. [9] We therefore set out to assess the readiness of PHCs in five selected states, each from one of the five geopolitical zones, respectively, that were not involved in the earlier study, to implement the multilevel strategy of the Hypertension Treatment in Nigeria Program. This assessment was conducted using an adapted version of the WHO’s Service Availability and Readiness Assessment (SARA) tool as part of a broader plan to scale up the program across other geopolitical zones in Nigeria.

## Method

### Study design

**Fig 1:**
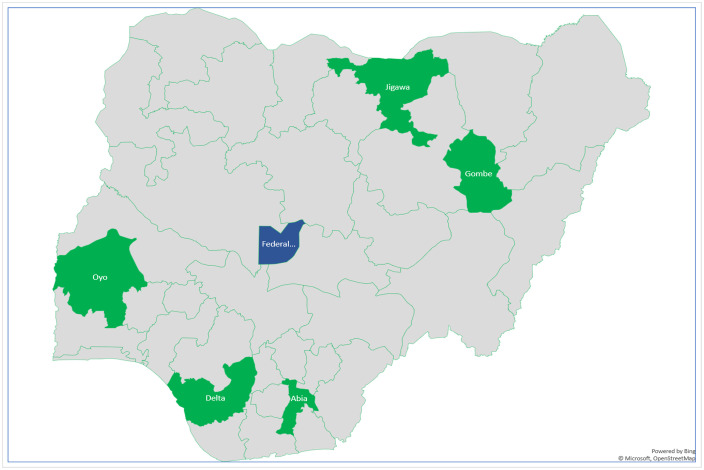
Map of Nigeria showing states that were selected from each of the geopolitical zones, respectively.

This cross-sectional survey was conducted nationwide from January to August of 2024. Of the six geopolitical zones in Nigeria. The first study was carried out in the Federal Capital Territory of Nigeria, located in the north-central geopolitical zone. In this study, using a multistage sampling technique, a state was randomly selected from each of the remaining five geopolitical zones (Fig 1): Oyo State (Southwest zone), Delta State (South-south zone), Abia State (Southeast zone), Jigawa State (Northwest zone), and Gombe State (Northeast zone). Isuikwuato and Umuahia North were chosen from among the seventeen local government areas (LGAs) in Abia State. Additionally, five PHCs were chosen from five villages in each of these LGAs, for a total of ten LGAs in the states. Other states likewise employed similar techniques, taking into account the facilities’ accessibility and geographic dispersion. The fifty PHCs from five states in the five geopolitical zones were evaluated using the same techniques used to assess PHCs in the Federal Capital Territory (Northcentral zone) in the first phase of the Hypertension Treatment Program in Nigeria [9].

This map of Nigeria displays the Federal Capital Territory of Nigeria, which is situated in the north-central geopolitical zone and was the site of the first study. A state from each of the remaining five geopolitical zones—Oyo State (Southwest zone), Delta State (South-south zone), Abia State (Southeast zone), and Jigawa State (Northwest zone)—was chosen at random for the study using a multistage sampling technique. Green—States randomly selected for the study. Blue—Federal Capital Territory.”

The PHCs included in the study were those being funded by the federal government, state government, local government, and some agencies, and also those being funded by the state government, local government, and other agencies without federal government involvement. Exclusion criteria were PHCs being funded by private organizations alone without input from the federal and/or the state government.

In summary, the fifty PHCs were evaluated using a modified version of the World Health Organization’s Service Availability and Readiness Assessment (SARA) instrument (S1 Table) [15]. The World Health Organization (WHO) Service Availability Readiness Tool is a globally recognized instrument utilized in many low- and middle-income countries. Our group has used it in the assessment of 60 healthcare facilities in the Federal Capital Territory in Nigeria and another 30 facilities in a state in the South-South region of Nigeria. It was adapted for use in our studies with input from the WHO Nigeria, the Non-Communicable Disease Division of the Federal Ministry of Health of Nigeria, and the Federal Capital Territory of Nigeria Primary Healthcare Board during stakeholders’ meetings. Its content validity was assessed by a panel of five experts in NCD care and primary healthcare. It was later pretested for clarity and relevance in five PHCs in the Federal Capital Territory, with minor revisions to the phrasing. The information obtained using this tool can help with the planning and management of the health system. There are three main domains: 1) service availability, 2) general service readiness, and 3) service-specific readiness and 13 sections, consisting of 1) service availability, 2) patient access, 3) staffing capacity, 4) infrastructure, 5) basic client amenities, 6) infection control, 7) healthcare waste management, 8) clinical mentoring, 9) basic equipment, 10) available services for non-communicable diseases and diagnostics, 11) supply chain, 12) medicines and vaccines, and 13) commodities [9,16].

Trained research assistants visited each of the PHCs to conduct interviews and site inspections using the SARA tool after obtaining written informed consent from the facility manager in each of the PHCs. Direct observation of activities in the PHCs and assessment of the equipment, drugs, and supplies available on the day of the interview were part of the SARA assessment procedure.

### Ethical approval

The study received ethical approval from the Federal Medical Centre Umuahia in Abia State, and it was accepted across the entire state. This approval was assigned the number FMC/QEH/G.596/Vol.10/730. Additionally, analogous approvals were obtained for the study in Delta, Gombe, Jigawa, and Oyo States.

Ethics committees of the five institutions that gave approval for the study in the five selected states, respectively, included the Jigawa State Ministry of Health (Jigawa State), Federal Medical Centre Umuahia (Abia State), Delta State University Teaching Hospital, Oghara (Delta State), Gombe State Ministry of Health (Gombe State) and Institute of Advanced Medical Research and Training, College of Medicine, University of Ibadan (Oyo State).

A consent document, written in plain English, that outlines the study’s purpose, procedures, duration, risks, benefits, confidentiality, right to withdraw, and the identity of the interviewer was presented to the participants for their review and verbal consent prior to the interview.

### Data quality assurance and control

To ensure data quality, field validation(s) and branching logic were developed and incorporated into the Research Electronic Data Capture (REDCap) software forms. Before obtaining data entry rights, all study team members with data access privileges completed the relevant training and passed a pilot test data entry using hypothetical data. Centralized statistical monitoring was performed through regular data and status quality reports. The data and status quality reports used the REDCap application programming interface functionality to export the program data and then were restructured and summarized using statistical software packages, including R (v4.0.3, R Core Team, Vienna, Austria) and SAS (v9.4, SAS Inc., Cary, NC). The reports were reviewed and discussed biweekly. Because the systems were in place and code had been generated from our team’s previous research, the reports were updated in near real time [17].

### Key indicators

Personnel were defined as reported full-time clinicians or paramedics, nursing professionals, pharmacists, laboratory technicians, community health extension workers, and community health officers. Service availability and readiness were defined as the proportion of amenities, equipment, diagnostic tests, or medicines within a defined domain of the data collection instrument.

### Statistical analyses

The study used graphical representations to assess the facility-based capacity and readiness for hypertension diagnosis and treatment. Continuous measures were summarized by median and interquartile range if non-parametrically distributed. Categorical measures were summarized as frequency and percent. Readiness and capacity were assessed based on domains of interest, including personnel, general service delivery, and cardiovascular service delivery in the hypertension treatment cascade; equipment and supplies; information systems; and blood pressure-lowering medications. Indicator scores for general and cardiovascular service readiness were calculated based on the proportion of sites with available amenities, equipment, diagnostic tests, or medicines within the SARA-defined domain. Data are presented descriptively. No comparative hypothesis tests (e.g., between states) were performed due to the formative, descriptive aims of this readiness assessment. For statistical analysis, the study team used SAS version 9.4 (SAS, Cary, NC, USA) and R version 3.5.1 (R Foundation, Vienna, Austria).

## Results

### Staff and service delivery

The findings of the facility officers’ interviews were used to tabulate the staffing level and service delivery in the five Nigerian states that were involved in the study (Table 1). Most PHCs (n = 43; 86%) reported having two or more full-time employees at the time of data collection. All ten (100%) PHCs chosen from Gombe State had reported having two or more staff, whereas 9 (90%) from Oyo State and 9 (90%) from Jigawa State had reported having two or more staff. Delta State had the least number of PHCs that reported having two or more full-time staff (n = 7; 70%).

**Table 1:** Capacity and Readiness Among Five States of Nigeria for Implementing a System-Level Hypertension Control Program within 50 Primary Healthcare Centres.

Site Characteristics	No. Sites Responded	All (n = 50)	Abia (n = 10)	Delta (n = 10)	Gombe (n = 10)	Jigawa (n = 10)	Oyo (n = 10)
Personnel and Training							
Sites with two or more full-time staff,^a^ n (%)	50	43 (86)	8 (80)	7 (70)	10 (100)	9 (90)	9 (90)
Number of full-time healthcare professionals, median (IQR)	50	6 (2 –9)	3 (2 –3)	3 (1 –4)	13 (10 –14)	7 (5 –12)	7 (2 –8)
Full-time community health extension workers, median (IQR)	50	2 (0-4)	1 (1 –2)	1 (0-2)	5 (4 –7)	3 (1 –4)	1 (0-2)
Full-time nurses, median (IQR)	50	1 (0-2)	1 (0-1)	1 (0-1)	2 (0-4)	3 (1 –4)	1 (0-2)
Full-time doctors (generalists and specialists), median (IQR)	50	0 (0−0)	0 (0−0)	0 (0-1)	0 (0−0)	0 (0−0)	0 (0−0)
Diagnose or manage NCDs	50	37 (74)	8 (80)	6 (60)	7 (70)	9 (90)	7 (70)
Diagnose or manage CVD	37	33 (89)	7 (88)	3 (50)	7 (100)	9 (100)	7 (100)
Received CVD training within the past two years, n (%)	33	8 (24)	1 (14)	0 (0)	3 (43)	1 (11)	3 (43)
Hypertension Service Delivery							
Screen for hypertension status, n (%)	50	49 (98)	10 (100)	10 (100)	10 (100)	9 (90)	10 (100)
Diagnose hypertension, n (%)	50	47 (94)	10 (100)	8 (80)	9 (90)	10 (100)	10 (100)
Confirm hypertension diagnosis, n (%)	50	42 (84)	10 (100)	8 (80)	6 (60)	8 (80)	10 (100)
Dispense initial treatment for hypertension, n (%)	50	38 (76)	4 (40)	6 (60)	9 (90)	10 (100)	9 (90)
Dispense follow-up treatment for hypertension, n (%)	50	33 (66)	3 (30)	1 (10)	10 (100)	9 (90)	10 (100)
Monitor patients with hypertension, n (%)	50	44 (88)	8 (80)	7 (70)	9 (90)	10 (100)	10 (100)
Provide long term care for patients with hypertension, n (%)	50	30 (60)	5 (50)	2 (20)	6 (60)	8 (80)	9 (90)
Equipment and Supplies for Hypertension							
Guidelines, n (%)	33	8 (24)	1 (14)	0 (0)	3 (43)	3 (33)	1 (14)
Treatment algorithms, n (%)	33	9 (27)	0 (0)	0 (0)	2 (29)	3 (33)	4 (57)
Information, education, and communication, n (%)	33	6 (18)	0 (0)	0 (0)	3 (43)	1 (11)	2 (29)
Functional blood pressure apparatus, n (%)	50	49 (98)	9 (90)	10 (100)	10 (100)	10 (100)	10 (100)
Information Systems							
Use of electronic patient records, n (%)	50	48 (96)	10 (100)	9 (90)	10 (100)	9 (90)	10 (100)
Functional landline phone, n (%)	50	12 (24)	1 (10)	0 (0)	4 (40)	3 (30)	4 (40)
Functional cellular phone, n (%)	50	29 (58)	0 (0)	3 (30)	9 (90)	7 (70)	10 (100)
Functional computer, n (%)	50	23 (46)	0 (0)	6 (60)	8 (80)	1 (10)	8 (80)
Access to email or internet, n (%)	49	21 (43)	0 (0)	4 (40)	5 (50)	7 (70)	5 (50)
Availability of Blood Pressure Lowering Medications							
Angiotensin Converting Enzyme Inhibitor, n (%)	50	18 (36)	1 (10)	0 (0)	7 (70)	7 (70)	3 (30)
Angiotensin Receptor Blocker, n (%)	50	7 (14)	0 (0)	0 (0)	3 (30)	2 (20)	2 (20)
Beta Blocker, n (%)	50	4 (8)	0 (0)	0 (0)	0 (0)	0 (0)	4 (40)
Calcium Channel Blocker, n (%)	50	36 (72)	4 (40)	3 (30)	10 (100)	10 (100)	9 (90)
Central acting agent, n (%)	50	19 (38)	0 (0)	2 (20)	3 (30)	8 (80)	6 (60)
Fixed Dose Combinations, n (%)	50	3 (6)	0 (0)	1 (10)	0 (0)	0 (0)	2 (20)
Diuretic,b n (%)	50	21 (42)	1 (10)	2 (20)	4 (40)	7 (70)	7 (70)
Vasodilator, n (%)	50	5 (10)	0 (0)	0 (0)	2 (20)	0 (0)	3 (30)
Number of 30-day treatment regimens in stock, median (IQR)	50	15 (0-132)	0 (0−0)	0 (0−0)	84 (15-475)	91 (19-561)	100 (26-330)
One or more 30-day treatment regimens in stock, n (%)	50	33 (66)	2 (20)	2 (20)	10 (100)	9 (90)	10 (100)

^a^Including all reported full-time clinicians or paramedics, nursing professionals, pharmacists, laboratory technicians, community health extension workers, and community health officers.

^b^Including furosemide, spironolactone, thiazide, or other diuretic.

CVD, Cardiovascular Disease; IQR, Inter-Quartile.

In addition, for the 50 PHCs, the median (interquartile range [IQR]) number of full-time health professionals was 6 (2 –9); community health extension workers (CHEWs) median = 2; IQR 0–4; full-time nurses median = 1; IQR 0–2; and full-time doctors (both specialists and generalists) median = 0; IQR 0–0.

### Hypertension service delivery

**Fig 2:**
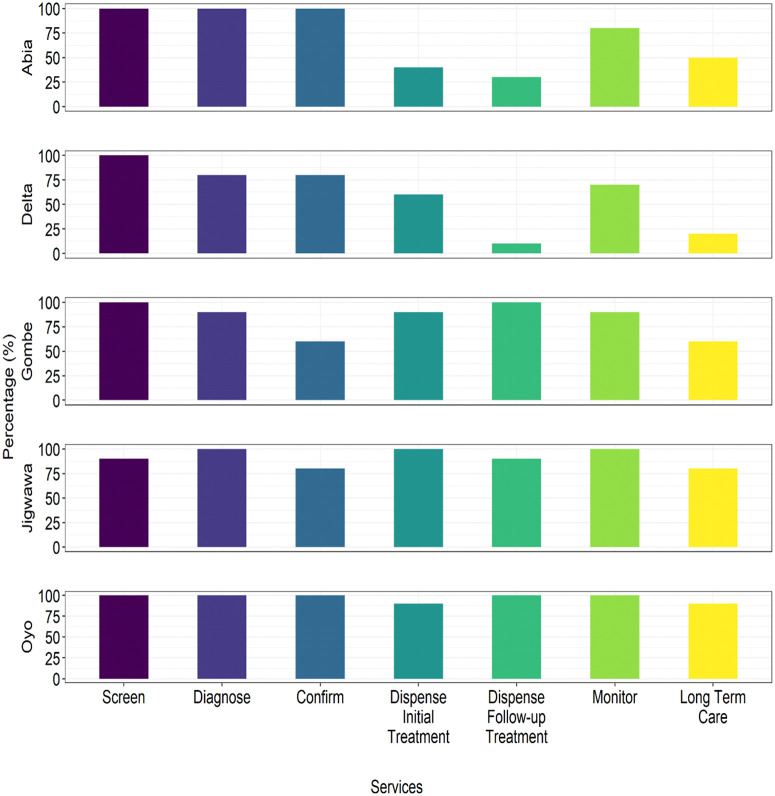
Cascade of hypertension services being provided by the PHCS in the states.

Out of the 50 PHCs assessed, 49 (98%), 47 (94%), and 42 (84%) were able to provide screening services for hypertension, diagnose hypertension, and confirm hypertension, respectively. Furthermore, the majority of PHCs were able to treat hypertension patients initially (n = 38; 76%), provide follow-up treatment (n = 33; 66%), monitor blood pressure (n = 44; 88%), and provide long-term care (n = 30; 60%). The ability to provide hypertension services was lowest in Abia and Delta States (Fig 2).

Fig 2 depicts the cascade of services for managing hypertension offered by the ten randomly chosen PHCs in each of the five states (Oyo, Jigawa, Gombe, Delta, and Abia) in Nigeria.

### Equipment and supplies for hypertension

The majority of the 50 PHCs, 49 (98%), had functional blood pressure apparatus, while only a minority of them, 8 (24%), had hypertension guidelines, treatment algorithms (n = 9, 27%), and information, education, and communication materials (n = 6, 18%). Abia and Delta States had the lowest proportions for equipment and supplies. Only 1 (n = 1, 10%) of the 10 PHCs in Abia State had guidelines, and none of the PHCs from Abia State had treatment algorithms, information, education and communication materials, or antihypertensive medication supplies. In Delta State, on the other hand, none of the 10 PHCs from the state had treatment guidelines, treatment algorithms, or information, education, and communication facilities and supplies for hypertension.

### Information systems

Twenty-three (46%) of the 50 PHCs had functional computers, with the highest numbers being in Gombe State and Oyo State (n = 8, 80%, respectively), followed by Delta State (n = 6, 60%). Conversely, Jigawa State (n = 1, 10%) and Abia State (n = 0, 0%) had the lowest computer availability. 0%) Twenty-one (43%) of the PHCs that responded to the question on availability of email or internet access had available internet, with the highest number reported in Jigawa State (n = 7, 70%), followed by Gombe and Oyo States (n = 5, 50%), and Delta State (n = 4, 40%). In Abia State, none of the ten PHCs had access to email or the internet.

### Availability of blood pressure-lowering medications

Thirty-three (66%) of the 50 PHCs that participated in the study had at least one 30-day treatment regimen in stock, while thirty-six (72%) had calcium channel blockers, twenty-one (42%) had diuretics, nineteen (38%) had central acting agents, eighteen (36%) had angiotensin converting enzyme inhibitors, seven (14%) had angiotensin receptor blockers, five (10%) had vasodilators, four (8%) had beta-blockers, and three (6%) had fixed-dose combinations. Additionally, the median number of 30-day treatment regimens in stock was 15 (IQR 0–132).

## Discussion

Fifty PHCs were chosen using a multi-stage random sampling approach from five states in Nigeria to participate in this cross-sectional national survey. The study looked at the PHC’s ability and site readiness for implementing a hypertension control program. The findings showed that there were shortcomings in the availability of blood pressure-lowering drugs, information systems, equipment and supplies for hypertension, personnel and training, and the provision of hypertension services.

### Personnel and training

Most PHCs had at least two full-time healthcare workers, primarily Community Health Extension Workers (CHEWs) and nurses, with a notable absence of full-time doctors. The staffing pattern does not align with the Minimum Service Package (MSP) requirements for PHCs in Nigeria, and the lack of doctors may impact the quality of medical care. However, the inadequate and inequitable distribution of healthcare workers in PHCs is not peculiar to Nigeria, as other studies around the world have reported similar findings [18–20].

However, the Hypertension Treatment in Nigeria Program was effective and implemented successfully with at least two full-time staff members, including solely non-physician healthcare workers like nurses and CHEWs. This index study serves as a formative evaluation for introducing other geopolitical zones of Nigeria to the successfully executed Hypertension Treatment in Nigeria Program in the Federal Capital Territory. [9] The two full-time employees notably satisfy the minimal requirement. Additionally, as stated in Nigeria’s National Task-shifting and Task-sharing (NTSTS) policy [21], the Federal Ministry of Health and Social Welfare has authorized the prescription of drugs for the prevention and control of NCDs by non-physician health workers in Nigeria, encouraging task-shifting and task-sharing with regards to NCDs management.

Additionally, a study conducted among Nigerian doctors revealed that more than half of the doctors had a favourable opinion of task sharing in the management of hypertension, with two-thirds supporting its use. [22] In addition, task shifting and task sharing have been proven effective in managing hypertension in the Hypertension Treatment in Nigeria Program, carried out in the FCT [9] and in some other studies in other LMICs. [23–25] Key strategies, such as expanded training, mentorship, and supportive supervision, had been reported as essential in enhancing the effectiveness of this approach in Nigeria [26,27].

### Hypertension service delivery

PHCs in Nigeria have a difficult time providing services for hypertension. Although 49 of the PHCs screened for hypertension, and 47 could diagnose the condition, only 42 (84%) of the PHCs in this index study verified the diagnoses, and only 30 (60%) offered long-term care. States like Abia and Delta showed less preparedness than Gombe, Jigawa, and Oyo in terms of the capacity to administer first medications (n = 38, 76%) and follow-up treatments (n = 33, 66%) for hypertension. Adopting a tailored service delivery paradigm, similar to successful HIV initiatives, may improve hypertension management in LMICs. [28,29] Strengthening PHC capacity by ensuring a consistent supply of essential medicines, enhancing staff training, and implementing standardized treatment protocols is crucial for effective hypertension control in Nigeria.^9, 10^. Indeed, the Hypertension Treatment in Nigeria Program has shown promising results, achieving treatment and control rates of over 90% and 50% through an integrated, multilevel care model within a 4-year period [7].

### Clinical guidelines and standardized treatment protocols

Thirty-five (70%) of the PHCs in this study had no hypertension clinical guidelines or treatment protocols, potentially leading to inconsistencies in hypertension management and consequent increased morbidity and mortality. This observation is not unique to Nigeria. Schutte et al., in a review of global disparities in blood pressure control, noted that the non-use of structured treatment algorithms contributes to significant variations in hypertension management across LMICs. [30] Standardized treatment protocols improve hypertension control, as exemplified by the HEARTS in the Americas initiative, which promotes implementing standard treatment protocols supported by a high-quality formulary, emphasizing rapid blood pressure control using two antihypertensive medications, preferably as a fixed-dose combination. [31] Indeed, a recent meta-analysis indicates that using standardized treatment protocols for hypertension is more effective than usual care, resulting in improved blood pressure control rates. [32] In response to these challenges, Nigeria has developed a simplified hypertension treatment guideline for primary healthcare, which, if widely adopted, could enhance treatment consistency and patient outcomes. The simplified treatment protocol was part of the implementation package of the successful Hypertension Treatment in Nigeria Program in the Federal Capital Territory, [9] and holds the potential for successful upscaling to other parts of the country.

### Digital infrastructure and information systems

Most PHCs’ inadequate digital infrastructure presents a significant challenge to healthcare delivery that requires attention. Less than half of the PHCs in this study had functional computers and internet access. This lack of basic infrastructure limits their ability to collect data, track patients, and provide continuous care. Communication was also limited, as only 12 (24%) PHCs had operational landlines. Digital health technologies can greatly improve healthcare access and efficiency in sub-Saharan Africa, particularly in remote areas. [33] A report from the WHO underscores the importance of investing in digital health solutions to support clinical decision-making and patient follow-up in LMICs. [34] Digital health solutions should be driven by local healthcare needs and supported by national governments and global organizations. [35] Mobile health applications, telemedicine, and electronic health records may enhance patient monitoring, treatment adherence, and provider decision-making. The District Health Information System 2 (DHIS2) has shown success in improving health information systems in low-resource settings. [36] Strengthening digital health systems like the DHIS2 in Nigerian PHCs is essential for optimizing hypertension management and healthcare delivery.

### Availability of blood pressure-lowering medications

Limited access to essential antihypertensive medications is a major barrier to effective hypertension management. In this study, essential antihypertensive medications were not commonly available. Calcium channel blockers were the most commonly available, but other classes, such as ACE inhibitors, ARBs, and diuretics, were in limited supply. Beta-blockers and vasodilators were scarcely available. Moreover, only 33 (66%) of PHCs had at least one 30-day treatment regimen in stock. An earlier publication in Nigeria corroborated these findings. ^10^ [10] Ineffective procurement procedures and high out-of-pocket costs worsen Nigeria’s insufficient access to medications [37].

Furthermore, disparities in medication availability across states—particularly in Abia and Delta, where stock levels are lowest—reflect broader challenges in drug distribution. In Nigeria, fragmented supply chains and inconsistent government funding lead to geographical disparities in medication access. [38] Weak pharmaceutical procurement systems within Nigeria’s PHCs contribute significantly to inadequate hypertension treatment and control. [9] Addressing these structural challenges is critical to ensuring equitable access to essential hypertension medications.

### Implications for policy and practice

These results highlight Nigeria’s critical need for targeted measures to improve basic healthcare for the management of hypertension.. Expanding training programs, enhancing information systems, and ensuring the availability of essential medications are critical steps to improving service delivery. Policies should address disparities in workforce distribution, medication supply chains, and digital infrastructure to achieve a more uniform and effective hypertension management system.

The Hypertension Treatment in Nigeria Program serves as a model for scaling up, demonstrating that multi-level strategies for hypertension diagnosis, treatment, and control can be effectively implemented in primary healthcare settings in African countries [9].

### Limitations

Our study had some limitations, which included the following:

(i) Of the more than 32,000 primary health centres in Nigeria, 50 were chosen for this study. Due to the modest size of our sample, it might not be an accurate reflection of all the PHCs in the country.
ii) Given that the study is cross-sectional, it does not account for the continuous advancements in manpower and facilities in Abia State and the other participating states.
iii) There was a possibility of selection bias despite random sampling due to a number of reasons, including the preference of some PHCs by the researchers and the distance of some of the PHCs. iv) The information provided in facility manager interviews may potentially be negatively impacted by the possibility of social desirability and information bias. The fact that our researchers confirmed the information provided by the managers, however, reduced the occurrence of this limitation.

## Conclusion

This study highlights critical gaps in workforce availability and training, hypertension service delivery, and availability of essential antihypertensive medications within Nigerian PHCs for hypertension management. While most PHCs had capacity for screening and diagnosis of hypertension, long-term care and medication availability were inadequate, with significant disparities across states. The reliance on non-physician health workers aligns with national task-sharing policies, but insufficient training remains a major barrier. Furthermore, the lack of use of standardized treatment protocols and inadequate digital infrastructure hinders effective hypertension control in Nigeria. Expanding staff training, expediting procurement procedures, guaranteeing a steady supply of pharmaceuticals, and fortifying digital health systems in PHCs are all essential to better managing hypertension in Nigeria. The federal, state, and local governments, as well as organizations that support PHCs, have responsibility for providing health-worker training, protocols for treating and controlling hypertension, and some measures to strengthen the essential medicine supply chain.

## Supporting information

S1 Table Service Availability and Readiness Assessment (SARA) | Implementation Guide, version 2.2 R. The table provides an overview of the survey’s steps and the activities to be undertaken at each step of a Service Availability and Readiness Assessment (SARA). Data should be generated and scheduled to align with the national health planning cycle. The length of time required to finish a SARA is determined by the size of the nation and whether a complete facility census is required. It usually takes three to six months to complete the procedure, from the first national customization of the assessment instrument to the data distribution and country report generation.
(DOCX)

S2 File List of collaborating investigators in the Hypertension Treatment in Nigeria Program,The names and affiliations of the collaborating authors of the Hypertension in Nigeria program who did not meet the inclusion requirements but made contributions in one way or another to the manuscript. (DOCX)
