# Supplementary material for: Capacity and site readiness for hypertension control program implementation in Nigeria: A nationwide cross-sectional study
Source: PLoS One. 2026 Mar 13;21(3):e0344011. doi: 10.1371/journal.pone.0344011 (PMC12987462; doi:10.1371/journal.pone.0344011)
Supplement: S1 Table — The table provides an overview of the survey’s steps and the activities to be undertaken at each step of a Service Availability and Readiness Assessment (SARA). Data should be generated and scheduled to align with the national health planning cycle. The length of time required to finish a SARA is determined by the size of the nation and whether a complete facility census is required. It usually takes three to six months to complete the procedure, from the first national customization of the assessment instrument to the data distribution and country report generation. (DOCX) [file pone.0344011.s001.docx]

**Service Availability and Readiness Assessment (SARA) | Implementation Guide, version 2.2 R**

| Steps | Survey activities |
| --- | --- |
| \| **1. Survey planning and preparation** \| \| --- \| | Establish a survey coordinating group of country stakeholders to oversee and facilitate the objectives, scope, design, implementation, and analysis.  • Obtain a list of all health facility sites (public, private, nongovernmental organizations (NGOs), and faith-based organizations (FBOs)), including country facility registry codes.  • Determine appropriate design methodology (census or sample), develop an implementation plan and budget, and secure funding.  • Review and adapt questionnaires to meet country-specific needs  • Recruit survey personnel (survey manager, field supervisors, data collectors, data entry/processing personnel, data analysts)  • Prepare a survey schedule  • Identify the survey sites (sampling frame). Select the sample size and sample of health facilities (if sampling methodology is chosen).  • Procure logistics, including equipment and transport, taking into consideration the number of sites to be visited, the number of data collection teams, drivers, vehicles, petrol, etc.  • Plan and conduct training courses for interviewers and field supervisors.  • Pilot test the survey in a selected number of health facilities, evaluate results, and make amendments if necessary. |
| **2. Data collection in the field** | Plan the data collection visits (prepare a letter of introduction, contact each site, and prepare a schedule of visits).  • Prepare materials and tools for data collectors  • Arrange for transport and regular communications during fieldwork.  • Assemble materials necessary for local data collection.  • Confirm appointments with health facilities  • Visit health facilities and collect SARA data in teams (usually two interviewers and a driver)  • At the end of the interview, check questionnaire and resolve missing/unreliable information  • Return completed forms and/or transfer electronic files to field supervisor at the conclusion of each day  • Return forms (paper and/or electronic) to survey manager when data collection is complete  • Conduct validation visits in surveyed sites (10%) to ensure quality of the collected data. |
| **3. Data processing, analysis and interpretation** | • Enter data using the CSPro application1 (on site or at the end of the day)  • Edit, validate and clean data set, check for consistency and accuracy  • Export the data set for analysis (SARA indicators)  • Conduct analyses of SARA data using the standard core indicators (SARA automated tool for results graphs and tables) as well as any country-specific indicators of interest  • Conduct analyses using data collected with the data verification and system assessment modules as well as a desk review of routine data available at the national level in view of the data quality review. |
| **4. Results dissemination** | • Meet with survey coordinating group to analyze and interpret survey results and to finalize recommendations  • Prepare the final report  • Plan and implement dissemination activities. The results should be used to support annual health reviews and feed into the M&E platform for the national health plan  • Document and archive the survey using metadata standards |

Sourced from Reference 15 (16 November, 2025).
