# Supplementary material for: Capacity and site readiness for hypertension control program implementation in Nigeria: A nationwide cross-sectional study
Source: PLoS One. 2026 Mar 13;21(3):e0344011. doi: 10.1371/journal.pone.0344011 (PMC12987462; doi:10.1371/journal.pone.0344011)
Supplement: S2 File — (DOCX) [file pone.0344011.s002.docx]

**S2: List of collaborating investigators in the Hypertension Treatment in Nigeria Program,**

Victor Davila Roman ^1^ Agomoh Princess Orieji ^2^ Eguono Marian Ebereghwa, ^3^ Chidubem Okoli, ^4^ Rabiu Garba, ^5^  Emmanuel Irobundu  ^6^  Lamkur Gabriel Shedul, ^6^ Nanna Ripiye, ^7^ Anthony Orji, ^7^, Grace Julcit Shedl^, 8^ Sam Osagie, ^9^ Tunde Ojo, ^9^ Confidence Joseph-Alo, ^9^ Charity Akor, ^9^ Rifkatu Reng,, ^9^ Angela Brown, ^10^ Zainab Mahmoud, ^10^ Guhan Iyer, ^10^ Valerie Graham, ^10^ Charles Goss, ^11^ Ming Cheng, ^11^ Mansi Agarwal, ^11^ Juliet Iwelunmor, ^12^ Julia Lopez, ^12^ Khaled Shorbaji, ^13^ Lisa Hirschhorn, ^14^ Abigail Baldridge, ^15^ Namratha Kandula, ^15^ Chisom Obiezu-Umeh, ^16^

**Collaborating investigators’ affiliations.**

1. Department of Medicine and Global Health Center, Washington University in St. Louis, Missouri, USA
2. Department of Public Health, Abia State Ministry of Health, Nigeria.
3. Department of Family Medicine, Delta State University Teaching Hospital, Oghara, Nigeria
4. Department of Public Health, University of Abuja Teaching Hospital, Abuja, Nigeria
5. Health Records Officers Registration Board of Nigeria (HRORBN), Dutse, Jigawa State
6. Community Medicine, University of Abuja Teaching Hospital, Abuja, Nigeria.
7. Family Medicine Department, University of Abuja Teaching Hospital, Abuja, Nigeria
8. Department of Pharmacy, University of Abuja Teaching Hospital, Abuja, Nigeria.
9. Cardiovascular Research Unit, University of Abuja. Department of Internal Medicine, University of Abuja Teaching Hospital, Abuja, Nigeria
10. Cardiovascular Division, Department of Medicine, Washington University, Missouri, USA
11. Statistics Department, Washington University in St. Louis, Missouri, USA
12. Infectious Disease Division, Internal Medicine Department, Washington University in St. Louis, Missouri, USA
13. Division of Biology & Biomedical Sciences. Washington University, Washington University in St. Louis, Missouri, USA
14. Department of Internal Medicine, School of Medicine, Northwestern University, Evanston, Illinois, United States.
15. Cardiology Division, Internal Medicine, Northwestern University, Evanston, Illinois, United States.
16. Implementation Science, Global Health, Northwestern University, Evanston, Illinois, United States.
